# Positioning tezepelumab for patients with severe asthma: from evidence to unmet needs

**DOI:** 10.1177/03000605241297532

**Published:** 2024-11-17

**Authors:** Carlo Lombardi, Cottini Marcello, Annamaria Bosi, Menzella Francesco

**Affiliations:** 1 Departmental Unit of Allergology, Clinical Immunology & Pneumology, Istituto Ospedaliero Fondazione Poliambulanza, Brescia, Italy; 2 Allergy and Pneumology Outpatient Clinic, Bergamo, Italy; 3 Pulmonology Unit, S. Valentino Hospital, Montebelluna (TV), AULSS2 Marca Trevigiana, Italy

**Keywords:** Asthma phenotype, endotype, alarmin, thymic stromal lymphopoietin, tezepelumab, biologics

## Abstract

Several endotypes of severe asthma with predominantly type 2 inflammation can be distinguished by the immune pathways driving the inflammatory processes. However, in the absence of type 2 inflammation, asthma is less clearly defined and is generally associated with poor responses to conventional anti-asthmatic therapies. Studies have shown that disruption of the epithelial barrier triggers inflammatory responses and increases epithelial permeability. A key aspect of this process is that epithelial cells release alarmin cytokines, including interleukin (IL)-25, IL-33, and thymic stromal lymphopoietin (TSLP), in response to allergens and infections. Among these cytokines, TSLP has been identified as a potential therapeutic target for severe asthma, leading to the development of a new biologic, tezepelumab (TZP). By blocking TSLP, TZP may produce wide-ranging effects. Based on positive clinical trial results, TZP appears to offer a promising, safe, and effective treatment approach. This narrative review examines the evidence for treating severe asthma with TZP, analyses clinical trial findings, and provides clinicians with practical insights into identifying patients who may respond best to this novel biologic therapy.

## Introduction

Several endotypes of severe asthma can be distinguished based on the immune pathways driving inflammatory processes. These are typically characterised by either predominant type 2 (T2) inflammation (termed T2-high), in which interleukin (IL)-4, IL-5, and IL-13 are key cytokines in the pathological processes, or by minimal to absent T2 inflammation (termed T2-low), driven by cytokines such as tumour necrosis factor alpha, IL-17A, and interferon gamma.^1,2^ In the absence of T2 inflammation, however, asthma is less clearly defined and lacks established biomarkers; it is also generally associated with poor responses to conventional anti-asthmatic therapies. Notably, endotype definitions of asthma remain controversial because interpretations of the relevance of specific pathways to a given molecular phenotype may change over time within the same patient.^3^

Chronic inflammation of the bronchial mucosa can undoubtedly induce structural changes in the airways, leading to airway remodelling marked by smooth muscle thickening and increased mucus production. Bronchial hyperresponsiveness (BHR) further contributes to excessive bronchoconstriction and restricted airflow. Environmental factors, both indoor and outdoor—collectively termed the exposome—are crucial in asthma pathogenesis, with genetic susceptibility significantly influencing disease onset and progression. The current understanding of the intrinsic and extrinsic factors that create a predisposition to asthma continues to grow, revealing a complex interplay between endosomal and exposomal influences in the pathophysiology of respiratory diseases, including allergies and asthma.^4^

Exposure to agents that damage barriers results in not only barrier disruption but also epithelial cell injury, colonisation by opportunistic pathogens, loss of commensal bacteria, reduced microbiota diversity, bacterial translocation, allergic sensitisation, and inflammation in the periepithelial area.^5^ Studies have shown that breaching the epithelial barrier triggers inflammatory responses and increases epithelial permeability, creating a vicious cycle that amplifies dysregulated subepithelial immune responses and enhances exposure to allergens and irritants.^6,7^ A key aspect of this process is the release of specific cytokines (IL-25, IL-33, and thymic stromal lymphopoietin [TSLP]) from epithelial cells in response to allergens and infection. These cytokines prime the immune system to generate both T2 and non-T2 immune responses, characterised by the activation of mast cells, eosinophils, dendritic cells, type 2 helper (Th2) cells, type 2 innate lymphoid cells (ILC2s), and neutrophils.^8^

TSLP is a pleiotropic cytokine, first described in 1994 as similar to IL-7, with a receptor that forms a heterodimer comprising the TSLP receptor and a common gamma-receptor chain alongside the IL-7 receptor.^9^ This member of the IL-2 cytokine family exists in short and long isoforms, with the long isoform involved in proinflammatory pathological processes.^10^ While TSLP is mainly secreted by epithelial cells in response to pathogenic stimuli, it is also produced by other cell types, including Th2 cells, dendritic cells, ILC2s, eosinophils, mast cells, stromal cells, fibroblasts, and macrophages. Numerous cell types express TSLP receptors, including haematopoietic progenitor cells, eosinophils, basophils, mast cells, airway smooth muscle cells, ILC2s, B and T lymphocytes, dendritic cells, neurons, and monocytes/macrophages.^11^ The extensive interaction of TSLP with both innate and adaptive immune systems underscores its significant capacity to initiate and sustain inflammatory responses in airway diseases. TSLP can also stimulate collagen production by fibroblasts and activate mast cells, promoting airway remodelling.^11^ TSLP has therefore been identified as a potential therapeutic target for severe asthma.

Tezepelumab (TZP) is a human monoclonal antibody immunoglobulin (Ig) G2λ that targets TSLP and represents the first biologic developed against an epithelial-derived cytokine. By blocking TSLP from binding to its receptors, TZP reduces TSLP-driven immune activation across various asthma endotypes. Clinical trials evaluating TZP in inflammatory allergic diseases have shown improvements in asthma exacerbation rates, inflammatory biomarkers, and lung function.^12^ Given TSLP’s extensive role within the immunologic network and TZP’s ability to counteract this activity, it is essential to identify patients who are most likely to benefit from TZP.

This narrative review therefore examines the evidence for TZP in treating severe asthma, analyses clinical trial findings, and provides clinicians with practical insights into the characteristics of patients who may respond best to this new biologic, especially given the diverse profiles of those eligible for this therapy.

## Methods

### Data source and study selection

An English-language literature review was conducted, focusing on clinical studies and reviews of TZP published up to and including July 2024. The biomedical databases consulted included MEDLINE (PubMed), SCOPUS, Google Scholar, EMBASE, and Web of Science. The main search terms used were ‘severe asthma’, ‘alarmins’, ‘TSLP’, ‘epithelial dysfunction’, ‘monoclonal antibodies’, ‘biologics’, ‘biomarkers’, ‘clinical trials’, ‘comorbidities’, ‘exacerbations’, ‘bronchial hyperresponsiveness’, and ‘lung function’.

This review was guided by the Scale for the Assessment of Narrative Review Articles (SANRA).^13^

## Tezepelumab for patients with T2 severe asthma: the evidence

In the PATHWAY series of randomised clinical trials, TZP significantly reduced the annualised asthma exacerbation rates (AAERs) across all three dosing regimens compared with placebo: 62% for the 70-mg dose, 71% for the 210-mg dose, and 66% for the 280-mg dose (p < 0.001 for all comparisons).^14^ These effects were observed regardless of the baseline blood eosinophil count (BEC) or the presence of other T2 inflammatory biomarkers. Additionally, all TZP groups showed substantial and sustained reductions in BEC and fractional exhaled nitric oxide (FeNO) levels, as well as progressive decreases in total serum IgE. These biomarker changes indicate that TZP has significant effects on the IL-4, IL-5, and IL-13 pathways, supporting the hypothesis that TSLP inhibition effectively targets both allergic and eosinophilic inflammation.

In the CASCADE double-blind, randomised, placebo-controlled, phase 2 trial, adult patients with uncontrolled moderate to severe asthma were randomly assigned (1:1) to receive either 210 mg of TZP or a placebo every 4 weeks for up to 52 weeks.^15^ The primary outcome measured was the change from baseline in the number of airway submucosal inflammatory cells (eosinophils, neutrophils, CD3+ T cells, CD4+ T cells, and mast cells) as assessed by bronchoscopic biopsy. The exploratory outcome was assessing BHR to a mannitol challenge. The reduction in airway submucosal eosinophils was 89% with TZP versus 25% with placebo, although no significant differences were observed between groups for other cell types. Airway BHR to mannitol was significantly reduced in the TZP group compared with placebo. These findings suggest that TSLP blockade with TZP may offer additional benefits in asthma management beyond reducing T2 airway inflammation.

**Figure 1.**
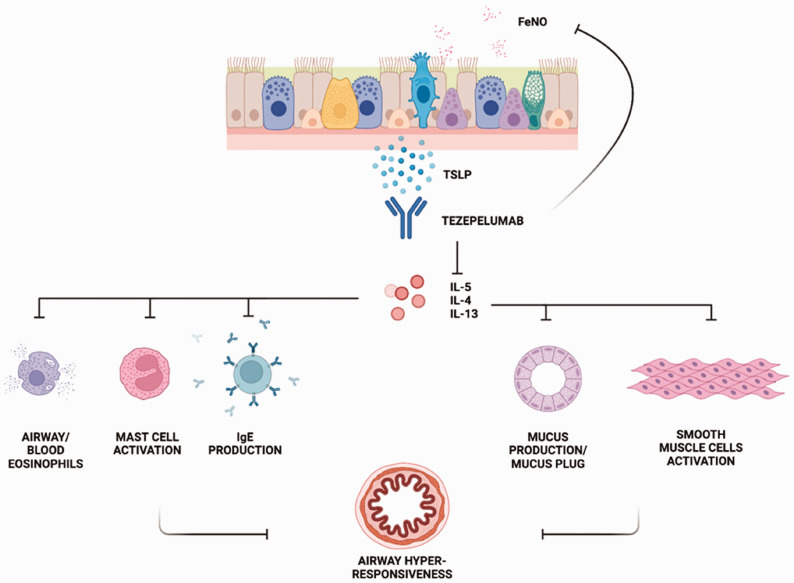
Tezepelumab mechanism of action. FeNO: fractional exhaled nitric oxide, TSLP: thymic stromal lymphopoietin, IgE: immunoglobulin E.

The NAVIGATOR study was a phase 3, multicentre, double-blind, placebo-controlled randomised trial involving 1061 adults and adolescents with severe asthma. The primary objective was to determine the AAER following subcutaneous administration of 210 mg of TZP every 4 weeks.^16^ The study population included roughly equal proportions of patients with high (≥300 cells/μL) and low (<300 cells/μL) BECs. TZP treatment resulted in reductions in the BEC, FeNO, and IgE levels, indicating that TZP may suppress multiple inflammatory pathways (Figure 1), potentially through decreased IL-5 and IL-13 levels.^17^ Furthermore, the reduction in total IgE levels could be linked to lowered IL-4 and IL-13 levels, leading to a progressive decrease in B-cell switching from IgM to IgE isotype production. These findings support the hypothesis that TSLP inhibition has broader physiological effects than simply targeting individual T2 cytokines.

Additional post hoc analyses of the NAVIGATOR study showed that TZP reduced the AAER over 52 weeks compared with placebo in patients eligible for omalizumab who had a BEC of at least 300 cells/μL.^18^ In a pooled analysis of PATHWAY and NAVIGATOR data, TZP reduced the AAER by 71% over 52 weeks compared with placebo in patients with a baseline BEC of at least 300 cells/μL and perennial aeroallergen sensitisation, indicating multiple drivers of inflammation.^19^

The SOURCE study was a phase 3 randomised controlled trial (RCT) designed to evaluate the corticosteroid-sparing effect of TZP in patients with severe OCS-dependent asthma.^20^ Although no statistically significant differences were found between the main study groups in terms of daily OCS dose reduction—the primary outcome—patients treated with TZP showed significant improvement when baseline BECs were ≥150 cells/μL and ≥300 cells/μL.

The DESTINATION phase 3 study was an extension of NAVIGATOR and SOURCE, aimed at evaluating the long-term safety and efficacy of TZP in individuals with severe, uncontrolled asthma.^21^ TZP reduced the AAER over 104 weeks compared with placebo; among participants from NAVIGATOR, the AAER ratio over 104 weeks was 0.42 (95% confidence interval [CI]: 0.35–0.51), and among those from SOURCE, it was 0.61 (95% CI: 0.38–0.96). A 68% reduction in AAER over 104 weeks was also observed with TZP compared with placebo in patients with a baseline BEC of at least 300 cells/μL and perennial aeroallergen sensitisation.^22^

Another notable aspect of TZP use in allergic patients is its potential synergy with allergen-specific immunotherapy. In a double-blind, placebo-controlled randomised trial involving patients with cat-allergic rhinitis, 121 participants were randomised to receive subcutaneous cat immunotherapy (SCIT), intravenous TZP, a combination of SCIT and TZP, or placebo for 52 weeks, followed by 52 weeks of observation.^23^ Nasal allergen challenge (NAC), skin testing, and blood and nasal samples were evaluated throughout the study. At week 52, the peak NAC-induced total nasal symptom scores (TNSSs) were significantly reduced in patients receiving TZP/SCIT compared with those receiving SCIT alone. By week 104—1 year after treatment cessation—the 0–1-hour area under the curve for TNSS, the primary outcome, was not significantly different between the SCIT and combined treatment groups. However, the peak 0–1-hour TNSS was significantly lower in those who received the combination therapy than in those who received SCIT alone. Transcriptomic analysis of nasal epithelial samples revealed that SCIT combined with TZP resulted in persistent downregulation of a gene network associated with T2 inflammation, correlating with improved NAC responses—an effect not observed with either monotherapy. These data suggest that TSLP inhibition by TZP enhances the efficacy of SCIT during treatment and may promote sustained tolerance after a 1-year treatment course.

The outcomes of the main studies on TZP are summarised in Table 1.

**Table 1.** Summary of main published studies on TZP

Trial	Patients	Duration of study	Main outcomes	Main results
PATHWAY(Corren et al., 2017)^14^	550 patients with moderate/severe asthma	52 weeks	AAER; increased prebronchodilator FEV_1_	Significant reduction of exacerbations vs. placebo, regardless of BEC countSignificant increase in FEV_1_
UPSTREAM(Sverrild et al., 2021)^66^	40 patients with asthma and AHR to mannitol	12 weeks	Change in PD15 to inhaled mannitol from baseline; change in airway inflammation	No significant change in AHR to mannitolReduction of airway inflammatory cells (eosinophils, mastocytes)
CASCADE(Diver et al., 2021)^15^	116 patients with moderate/severe asthma	52 weeks	Change in number of airway submucosal inflammatory cells in bronchoscopic biopsy sample from baseline to end of treatment; AHR to mannitol evaluation	Significant reduction of submucosal eosinophilsReduction of AHR to mannitol
NAVIGATOR (Menzies-Gow et al., 2021)^16^	1061 patients with severe uncontrolled asthma	52 weeks	AAER vs. placebo	Significant reduction of exacerbations vs. placebo
SOURCE(Wechsler et al., 2022)^20^	150 patients with asthma treated with high doses of ICS and OCS	48 weeks	Reduction of OCS dose	No significant reduction of OCS use in the overall population; a significant improvement in the OCS sparing effect only in TZP-treated patients with a baseline BEC of >150 cells/μL
DESTINATION (Extension study)(Menzies-Gow et al., 2023)^21^	1061 (NAVIGATOR) + 150 (SOURCE) patients	104 weeks	Adverse events; reduction of AAER	No significant rate of side effects vs. placebo; significant reduction of exacerbations vs. placebo

RCT: randomised controlled trial, FEV_1_: forced expiratory volume in 1 second, BEC: blood eosinophil count, AHR: airway hyperresponsiveness, PD15: provocative dose causing a 15% decrease in FEV_1_, OCS: oral corticosteroids, AAER: annualised asthma exacerbation rate.

## Tezepelumab for patients with non-T2 severe asthma: the evidence

Non-T2 asthma is generally characterised by neutrophilic or paucigranulocytic inflammation and typically responds poorly to inhaled corticosteroids.^24^ It is frequently associated with factors such as obesity, smoking, pollutants, advanced age, and viral or bacterial infections,^25^ and is most frequently defined by low BEC and/or low FeNO levels. Clinical evidence from the PATHWAY, CASCADE, and NAVIGATOR studies, as well as pooled data from PATHWAY and NAVIGATOR, show that TSLP inhibition with TZP reduces the AAER compared with placebo—independently of BEC or other Th2 biomarkers—and improves clinical outcomes among patients with uncontrolled asthma receiving long-acting β2 agonists and medium or high doses of inhaled glucocorticoids.^19,26^ In NAVIGATOR, for instance, reductions in exacerbations with TZP ranged from 37% in the T2-low severe asthma subgroup (BEC < 300 cells/μL and FeNO < 25 ppb) to 77% in the T2-high severe asthma subgroup (BEC ≥ 300 cells/μL and FeNO ≥ 25 ppb). Notably, TZP reduced the annualised rate of severe exacerbations requiring emergency department visits or hospitalisation by 79% in the overall population and by 60% to 90% in the T2-low subgroup relative to the T2-high subgroup.^27^

**Figure 2.**
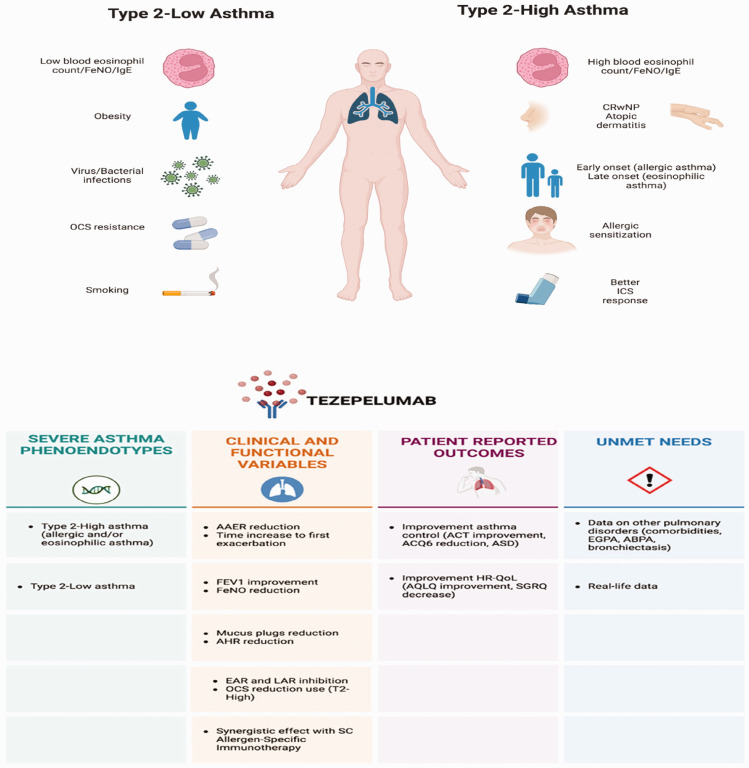
Main characteristics and outcomes of patients potentially responsive to tezepelumab IgE: immunoglobulin E, FeNO: fractional exhaled nitric oxide, CRSwNP: chronic rhinosinusitis with nasal polyposis, ICS: inhaled corticosteroids, AAER: annualised asthma exacerbation rate, FEV_1_: forced expiratory volume in 1 second, AHR: airway hyperresponsiveness, ACQ: Asthma Control Questionnaire, ASD: asthma symptom diary, AQLQ: Asthma Quality of Life Questionnaire, SGRQ: St. George’s Respiratory Questionnaire, SC: subcutaneous, EAR: early responses, LAR: late responses, EGPA: eosinophilic granulomatosis with polyangiitis, ABPA: allergic bronchopulmonary aspergillosis.

These findings underscore the potential advantages of targeting an upstream cytokine, such as TSLP, that can broadly influence disease activity rather than focusing on a single downstream pathway or specific cell target (Figure 2). Overall, data from TZP clinical trials in patients with non-T2 characteristics suggest that TZP, while somewhat less impactful than in T2 populations, presents a promising therapeutic option for a large group of patients with severe asthma who previously had limited treatment options, including those ineligible for any of the currently approved monoclonal antibodies.

## Tezepelumab and body mass index (BMI)

A post hoc analysis evaluated the long-term efficacy of TZP in patients from the NAVIGATOR and DESTINATION studies, stratified by baseline BMI.^28^ In the placebo group, the AAER over 104 weeks was higher among those with a higher BMI. In the TZP group (210-mg dose), the AAER over 104 weeks decreased compared with placebo by 58% in patients with a BMI below 25 kg/m^2^, by 59% in those with a BMI between 25 and 30 kg/m^2^, and by 57% in those with a BMI of 30 kg/m^2^ or greater (95% CI: 42–68). This indicates that 210-mg doses of TZP reduced asthma exacerbations over 2 years, regardless of baseline BMI. These findings further demonstrate TZP’s efficacy across a broad population of patients with severe, uncontrolled asthma, including those with BMIs in the healthy, overweight, and obese ranges.

## Tezepelumab: addressing unmet needs

### Severe asthma and comorbidities

Severe asthma is often accompanied by various comorbidities that complicate disease management, affect patient outcomes, contribute to poor disease control, and can mimic asthma symptoms.^29^ These comorbidities are more prevalent in severe asthma than in mild to moderate cases and can be categorised as either pulmonary or extrapulmonary. Pulmonary comorbidities include upper respiratory tract disorders such as allergic and nonallergic rhinitis, chronic rhinosinusitis with nasal polyposis (CRSwNP), and obstructive sleep apnoea, as well as middle/lower respiratory tract disorders such as chronic obstructive pulmonary disease, allergic bronchopulmonary aspergillosis, and bronchiectasis. Extrapulmonary comorbidities include anxiety, depression, gastro-oesophageal reflux disease, obesity, and cardiovascular and metabolic diseases.

CRSwNP is particularly severe in patients with comorbid asthma, characterised by high rates of nasal polyp recurrence and greater corticosteroid dependence compared with asthma alone (4% vs. 1% of patients).^30,31^ RCTs have shown favourable effects of biological agents such as dupilumab, mepolizumab, and omalizumab on CRSwNP,^32,33^ and studies have also revealed positive outcomes in patients with both severe asthma and CRSwNP when treated with these biologics.^34–36^ Ongoing studies are now evaluating the effect of TZP in patients with CRSwNP, with findings to be compared with those of other biologics. Specifically, the WAYPOINT multicentre, randomised, double-blind, parallel-group, placebo-controlled phase 3 trial is currently assessing the efficacy and safety of TZP in severe CRSwNP.^37^ More than 400 participants are enrolled in a 52-week treatment period followed by a 12- to 24-week post-treatment follow-up for those completing the initial treatment, with an estimated study completion date of December 2024.^38^

Data on the impact of TZP in patients with both severe asthma and CRSwNP are already promising. A post hoc analysis from the phase 3 NAVIGATOR study evaluated TZP’s effect on Sino-Nasal Outcomes Test (SNOT-22) scores in patients with a history of nasal polyposis (NP).^39,40^ Changes from baseline to week 52 were assessed in patients with reported NP, categorised based on whether the NP occurred within 2 years prior to randomisation. Mean baseline SNOT-22 scores were similar across patients with an NP history; for those receiving TZP, the change from baseline was 21.06, compared with 10.48 in those receiving placebo. At week 52, TZP treatment led to improvements across all five SNOT-22 domain scores, with the most notable gains in the sleep, function, and nasal domains. These findings indicate that TZP provided clinically meaningful improvements in rhinosinusitis symptoms over 52 weeks compared with placebo in patients with severe, uncontrolled asthma and a history—whether recent or remote—of comorbid NP.

A post hoc analysis of the PATHWAY study (ClinicalTrials.gov number NCT02054130) further assessed TZP’s efficacy in adults with severe, uncontrolled asthma, with and without NP.^41^ TZP reduced the AAER compared with placebo to a similar extent in patients with and without NP (75% and 73% reduction, respectively). Patients receiving TZP showed greater reductions in the BEC and in the FeNO, IL-5, and IL-13 levels compared with those on placebo, regardless of the NP status. These results suggest that TZP effectively reduces exacerbations and inflammatory biomarkers in patients with or without NP, supporting its use in a broad population of patients with severe asthma. Additionally, a case report detailed how TZP treatment improved asthma symptoms, nasal obstruction, and olfactory dysfunction in an elderly Japanese woman with aspirin-exacerbated respiratory disease and CRSwNP.^42^

### Asthma remodelling and mucus plugs

Epithelial cytokines, including TSLP, play a role in airway remodelling in asthma, and targeting them may help disrupt this process; thus, studies evaluating TZP’s impact on remodelling are valuable. This focus aligns with a recent re-evaluation of the concept of complete asthma remission and the role of anti-asthmatic therapy in patients with mucus plugs. Mucus plugs are common in asthma, occurring in approximately 60% of patients, and tend to persist over time,^43–45^ posing risks across all stages of asthma severity. Mucus plugs exacerbate airway obstruction due to smooth muscle-mediated bronchoconstriction, while excessive mucus leads to impaired ventilation, persistent obstruction, hospitalisation, and potentially fatal airway plugging.^46,47^ Current asthma treatments, however, do not directly target mucus hypersecretion. Of particular interest is the finding from the CASCADE RCT that TZP was associated with a reduction in occlusive mucus plugs compared with placebo in patients with moderate to severe uncontrolled asthma. In this study, the absolute change from baseline in mucus plug scores was −1.7 ± 2.6 in TZP-treated patients (n = 37) compared with 0.0 ± 1.4 in placebo-treated patients (n = 45).^48^ This effect is likely due to TSLP’s broad influence as a pleiotropic cytokine acting on multiple cell types involved in mucus plug formation. Further studies investigating TZP’s impact on mucus plugs are warranted.

### Seasonal exacerbations

Despite significant advancements in preventing exacerbations, recurrent asthma flare-ups remain a persistent issue.^49^ Viral respiratory infections are known to be the most common triggers, accounting for 50% to 80% of asthma exacerbations in adults.^50^ Human rhinoviruses, especially types A and C, are particularly common^51,52^ and are closely linked to children returning to school and the so-called ‘September epidemics’.^53^ The combination of allergic sensitisation and viral infections considerably heightens the risk of exacerbation, even in severe asthma, while exposure to both perennial and seasonal allergens is another key risk factor.^54,55^ A post hoc analysis of NAVIGATOR assessed TZP’s effect on asthma exacerbations across seasons^56^ and showed that TZP reduced the AAER compared with placebo by 63% (95% CI: 52%–72%) in winter, 54% (95% CI: 41%–64%) in autumn, 46% (95% CI: 26%–61%) in spring, and 62% (95% CI: 48%–73%) in summer. For patients with seasonal allergies, TZP reduced the AAER compared with placebo by 59% (95% CI: 29%–77%) during spring and by 70% (95% CI: 33%–87%) during the ragweed season in September. In patients sensitised to perennial allergens, TZP reduced the AAER compared with placebo consistently across all seasons.

### Comparison with other biologics, with a focus on dupilumab

The majority of subjects enrolled in RCTs for severe asthma have T2-high endotypes. However, from the outset of TZP’s clinical development, patients with T2-low endotypes have also been included to address a significant unmet need. Selection for trial enrolment has primarily been based on the underlying endotype and phenotype,^57^ with the rate of asthma exacerbations as the main criterion, followed by OCS dependence. Secondary parameters include respiratory function, symptom control, and reliever use. The selection process for biologics has varied based on specific markers: atopy has guided anti-IgE RCTs, eosinophilic inflammation has informed mepolizumab and benralizumab trials, and T2-high inflammation has been the primary criterion for dupilumab.^58^

When comparing the mechanisms of action of various monoclonal antibodies, dupilumab is the most similar to TZP, particularly in terms of patient eligibility. In the LIBERTY ASTHMA QUEST study, baseline exacerbations averaged 2.0 per year before treatment with dupilumab, which aligns with the mean of 2.0 in the NAVIGATOR study before TZP treatment.^16,59^ Dupilumab reduced the exacerbation rate by 47% overall and by 67% in patients with BECs of at least 300 cells/μL.^59,60^ Similarly, TZP achieved a 50% overall reduction in exacerbations in NAVIGATOR, with reductions reaching up to 70% in patients with BECs of at least 300 cells/μL, where eosinophilia was the most predictive biomarker for a favourable response.

Regarding OCS use, in the LIBERTY ASTHMA VENTURE study of dupilumab, the mean baseline dose of OCS was 11 mg, while in the SOURCE study of TZP, it was 11.8 mg.^20,60^ Both biologics achieved a mean reduction of 50% from baseline, although the SOURCE study did not meet its primary outcome except in patients with baseline BECs over 150 cells/μL, who demonstrated a significant OCS-sparing effect.^20^ New studies, including WAYFINDER (NCT05274815) and SUNRISE (NCT05398263), aim to address these limitations. Preliminary results from WAYFINDER indicate that the maintenance OCS dose was reduced from 10.9 mg/day to 5 mg/day for over 90% of enrolled patients.^61^

Across RCTs, dupilumab showed the greatest improvement in respiratory function, with an average increase in forced expiratory volume in 1 second of approximately 300 mL, followed by TZP at approximately 230 mL. TZP also demonstrated promising results in reducing BHR, a unique outcome not directly comparable to other biologics because they have not been studied for this variable.^58^

### Safety profile

Pooled safety data from the main RCTs indicate lower rates of adverse events—including those leading to treatment discontinuation and other serious and non-serious events—in patients treated with TZP compared with placebo.^62^ A network meta-analysis similarly found no significant difference in the incidence of adverse events between TZP and placebo groups.^57^ The most common adverse events reported were asthma, nasopharyngitis, headache, upper respiratory tract infection, and bronchitis.

The long-term DESTINATION extension study evaluated TZP’s safety and efficacy in adults with uncontrolled asthma over 104 weeks.^21^ In this study, serious thoracic, respiratory, and mediastinal issues occurred less frequently in TZP-treated patients than in the placebo group. However, a higher incidence of cardiac adverse events classified as *serious* was observed in patients receiving TZP compared with those on placebo. Importantly, the *overall* incidence of cardiac adverse events, of cardiovascular adverse events classified as *major*, and of *deaths* from cardiovascular causes were similar between the TZP and placebo groups, suggesting no direct correlation with the drug. Nonetheless, continued evaluation of cardiac events in ongoing and future studies remains important.

## Conclusions

Upstream inhibition of TSLP may provide a broad anti-inflammatory effect,^62^ and the new, safe, and promising approach with TZP appears to offer effective treatment across a wider and more diverse patient population with varying phenotypes (Figure 2). TZP is highly effective in severe allergic and eosinophilic asthma and shows potential for use in severe asthma cases where common T2 biomarkers are within normal ranges. The development of tests for specific biomarkers of TSLP-mediated inflammation could further enhance the selection of patients most likely to benefit from TZP.

Based on current studies, TZP can expand the range of severe asthma patients eligible for biologic treatment, extending its use beyond the T2-high phenotype to include patients with T2-low and overlapping phenotypes. The presence of BHR or mucus plugs, along with TZP’s favourable therapeutic response in overweight and obese patients without the need for dose adjustments, are also factors to consider when selecting TZP.^57^ The ideal candidate profile for TZP, regardless of BEC, is a patient aged 12 to 80 years with severe allergic and eosinophilic asthma who experiences exacerbations despite high doses of inhaled corticosteroids, alone or with long-acting β2 agonists. According to the existing literature, TZP’s strongest responders are patients with multiple inflammatory drivers, such as those with documented aeroallergen sensitisation and eosinophilia or those with high FeNO levels, BHR, or mucus plugs. However, patients with low eosinophil or FeNO levels may also benefit from TZP, though to a lesser degree.^63–65^ Additionally, TZP has been shown to reduce seasonal exacerbations in patients with severe, uncontrolled asthma, effectively mitigating flare-ups across seasons for patients with viral triggers as well as those with seasonal and perennial allergies. These findings further support TZP’s efficacy in a broad population of patients with severe, uncontrolled asthma (Figure 2).^56^

Finally, several issues remain to be clarified. Although this new biologic with its innovative therapeutic target appears to offer effective treatment to a broader and more diverse patient population than current options, specific biomarkers for TSLP-mediated inflammation are not yet available; such biomarkers would enable more precise selection of ideal candidates. Identifying patients with BHR could also be beneficial, though challenges remain in assessing BHR with simple and safe tests in individuals with severe asthma. Further research is needed on the role of alarmins in asthma pathogenesis, which could enhance understanding of the immunological pathways involved in various endotypes and phenotypes.

Another current limitation is the small amount of data on TZP in patients who have severe asthma with comorbidities, such as CRSwNP and bronchiectasis. Additional double-blind, placebo-controlled studies are essential to refine TZP’s role in future indications, which would enhance the selection of the most suitable treatments given the expanding range of therapeutic options. Furthermore, real-world studies and head-to-head prospective trials would provide valuable insights to improve the application of novel biologic agents. Finally, identifying optimal pathways for biologics in non-responders is critical for making informed therapeutic adjustments, including from a pharmaco-economic perspective, even before considering previously approved or upcoming monoclonal antibodies as secondary treatment options.

## Data Availability

Data sharing is not applicable to this article because no datasets were generated or analysed during the current study.
