# Corrigendum to “Positioning tezepelumab for patients with severe asthma: from evidence to unmet needs”

**DOI:** 10.1177/03000605241306931

**Published:** 2024-12-05

Lombardi C, Marcello C, Bosi A, et al. Positioning tezepelumab for patients with severe asthma: from evidence to unmet needs. *J Int Med Res* 2024; 52(11). doi:10.1177/03000605241297532

The first and last names of the second and fourth authors were inadvertently swapped in the article. The correct names are ‘Marcello Cottini’ for the second author and ‘Francesco Menzella’ for the fourth author.

The article has been corrected online.

